# Addiction to B-MYB

**DOI:** 10.18632/oncotarget.132

**Published:** 2010-08-04

**Authors:** W. Clay Gustafson, William A. Weiss

**Affiliations:** ^1^Department of Pediatric Hematology/Oncology, University of California San Francisco, CA94158, USA; ^2^Department of Neurology, University of California San Francisco, CA 94158, USA

Neuroblastoma, the most common and deadly extracranial solid tumor of childhood, has long provided a challenge to both clinicians and basic scientists. Despite its high prevalence among pediatric cancers and extensive efforts over the last several decades, there is still been only marginal improvement in overall survival and almost no improvement in survival for high-risk neuroblastoma [1]. Numerous risk stratification studies have shown that amplification of the *MYCN* oncogene is the clearest clinical indicator of poor prognosis.

Despite the importance of *MYCN* amplification in neuroblastoma, little work has been done to examine the transcriptional regulation of the *MYCN* amplicon. In addition to transcriptional regulation, a second primary means through which cells regulate levels of Mycn is at the level of protein stability. Therefore, exploring methods enabling targeting of Mycn protein stability is a major focus of current therapeutic research (reviewed in [2]). If significant transcriptional regulation of the *MYCN* amplicon in neuroblastoma could be characterized and exploited, these could result in novel strategies to target this deadly disease.

What do we know about transcriptional regulation of MYCN in neuroblastoma? In conjunction with binding partners including Max and Miz, Myc family transcription factors (including *c-MYC*, *MYCN*, and *MYCL*) bind to specific “E-box” elements to regulate gene expression. E-boxes are quite common in the genome and are present in the promoter regions of approximately 25% of known genes indicating complex and tissue specific regulation of Myc mediated transcription [3]. While many downstream transcriptional targets of c-Myc have been determined (including the B-MYB gene)[4], detailed analysis and confirmation of MYCN regulated genes is of vital importance to our understanding of MYCN mediated oncogenesis. In this issue of Oncotarget, Gualdrini et al propose the *B-MYB* transcription factor as one such target, and provide preliminary evidence for a reciprocal regulation between the amplified *MYCN* and the B-MYB transcription factors [5].

Expression of the B-MYB transcription factor has been previously shown to be a poor prognostic indicator in neuroblastoma, independent of *MYCN* amplification [6]. In this study, Gualdrini et al show an intriguing correlation between expression of mRNA for MYCN and B-MYB in neuroblastoma patient samples amplified for *MYCN*, extending this observation to the protein level in several *MYCN* amplified neuroblastoma cell lines. Importantly, the correlation between MYCN and B-MYB does not hold true in MYCN non-amplified samples. Additionally, using CHIP in established cell lines, the authors provide preliminary evidence that Mycn protein can bind to and induce transcription of B-MYC, consistent with B-MYB as a transcriptional target of MYCN. Perhaps most importantly, the study shows that knockdown of B-MYB by shRNA blocks proliferation in neuroblastoma cell lines with amplified *MYCN*. However, mechanistic details of this synthetic anti-proliferative effect between knockdown of B-MYB and amplification of *MYCN* remain to be determined. Also importantly, the authors use a single shRNA against BMYB, and do not extend this observation to primary tumors, thus limiting the impact of this result. Lastly, B-MYB was also shown to bind to the *MYCN* promoter in chromatin-IP assays of *MYCN* amplified neuroblastoma cells in culture, and induced a relatively modest transcriptional upregulation of a *MYCN* promoter element.

The authors use these exciting albeit preliminary results to propose a novel model for a reciprocal regulatory loop between these two transcription factors. If significant epigenetic and transcriptional regulation of amplified *MYCN* (implied by these studies) can be validated in primary tumors, these studies would support BMYB as target for therapeutic intervention in *MYCN* amplified cancers. However, given myriad difficulties currently in-place in targeting Mycn itself, the ability to target both BMYC and MYCN in neuroblastoma raises its own group of challenges.
